# Safety and Efficacy of Three Modifications of Canaloplasty to Treat Open-Angle Glaucoma: 3-Year Outcomes

**DOI:** 10.3390/jcm12206475

**Published:** 2023-10-11

**Authors:** Aleksandra K. Kicińska, Marek Rękas

**Affiliations:** Ophthalmology Department, Military Institute of Medicine—National Research Institute, 04-141 Warsaw, Poland; rekaspl@gmail.com

**Keywords:** mini-canaloplasty, ABiC, uncontrolled primary open-angle glaucoma, phaco-canaloplasty

## Abstract

Background: This is a report of 3-year results of a prospective assessment of three modifications of canaloplasty (C): ab externo (ABeC), mini-canaloplasty (miniABeC), and ab interno (ABiC) performed concomitantly with cataract removal in subjects suffering from primary open-angle glaucoma (POAG). Methods: Forty-eight individuals were randomized for one of the surgeries: ABeC, miniABeC, or ABiC and cataract removal—16 eyes for each group. Follow-up examinations were carried out on the day of the surgery, on days 1 and 7, after 1, 3, 6 months, and at 1, 2, and 3 years. Complete and qualified success was an IOP ≤ 15 mmHg without or with antiglaucoma eye drops, respectively. The IOP reduction of 20% or more was considered an additional success criterion. Results: Within three years the probability of qualified success was ABiC and miniABeC—94%, ABeC—100%, and of complete success ABiC—75%, miniABeC—100%. At the 3-year follow-up, the median IOP decreased from 22 to 15 mmHg in the ABeC group (*p* = 0.001), from 22 to 15 mmHg in the miniABeC group 15 (*p* < 0.001), and from 21 to 15 mmHg in the ABiC group (*p* = 0.001) compared to the post-washout stage. The IOP dropped by 20% or more without medications in 56.2% of patients post ABiC, 68.8% post miniABeC and 75% post ABeC. The median number of antiglaucoma medications dropped in all three groups; at the 3-year follow-up, only one patient following ABeC and four subjects following miniABeC required treatment. One patient required reoperation and further intensification of topical treatment—post miniABeC. The levels of IOP, CDVA, and success probability at the 36-month follow-up showed no significant difference for individual groups. Conclusions: ABeC, miniABeC, and ABiC have significant IOP-lowering potential in individuals diagnosed with POAG at a mild to moderate stage and no history of IOP ≥ 30 mmHg with a good safety profile.

## 1. Introduction

Currently, there are many surgical options for the treatment of patients suffering from primary open-angle glaucoma (POAG) aimed at controlling intraocular pressure (IOP). While trabeculectomy (TC) is still considered the golden standard, it has a noticeable complication rate and requires bleb-preserving procedures, which seriously affect patients’ quality of life. These drawbacks urged a search for safer alternatives with satisfactory IOP-lowering potential. In the 1990s, Stegmann performed viscocanalostomy (VC) in the African population with good results, and Schlemm’s canal surgery was born [1]. The rationale behind procedures of this type is based on the findings of W. Morton Grant, who established that up to one-fourth of outflow resistance was localized in the outer wall of the SC (SCOW) and peripherally from it [2]. However, VC or SC implants only act at the site of the incision, thus reducing only some amount of distal resistance. Canaloplasty (C), on the contrary, involves 360 degrees of the SC’s circumference and has additional impact on the SCOW and the trabecular meshwork (TM), and thus gained a lot of interest. Its action is based on three principles: viscodilation of SC, tensioning of the TM, and aqueous percolation through the trabeculo-Descemet window (TDW). It is still not known to what extent each of them is important. Also, the exact role of the intrascleral space and its effect on the distal outflow pathway have not been determined yet.

The multifactorial action of the surgery and the unique design of the iTrack microcatheter caused extensive exploration of its potential, and many modifications of ab externo canaloplasty (ABeC) emerged. In order to enhance IOP reduction, the conventional technique was combined with extra suprachoroidal drainage outflow [3,4] or an additional canal expander [5]. To minimize costs, cathetherless C was attempted with a suture probe instead [6]. In search of less incisional and more conjunctiva-sparing variants, ab interno canaloplasty (ABiC) [7] and mini-canaloplasty (miniABeC) [8] gained popularity as micro- and minimally invasive techniques. What is more, C has been considered as useful in gene therapy for POAG [9] or telemetric IOP self-monitoring with a special sensor [10] in animal models.

The treatment of POAG is life-long and thus requires strategic thinking about the patient’s health and vision. Blebless procedures enable the preservation of the tissues for future surgeries if needed. Less invasive procedures have a good safety profile, so they can be repeated, which makes them an alternative in patients with ocular surface disease or noncompliant to topical treatment. Previously, the authors published 1-year results in this prospective study concerning a comparison of modifications of C: ABeC, miniABeC, and ABiC [11]. All three surgical techniques gave a significant lowering of IOP and the amount of antiglaucoma medications with few complications and did not present significant differences when considering both safety and effectiveness. The present article refers to the stability of those results.

## 2. Patients and Methods

### 2.1. A Study

This article is a 3-year report and a continuation of a prospective study on C carried out by the first author. The principles of the Declaration of Helsinki were abided and acceptance from a Bioethics Committee was obtained. The Clinical Trials Identifier is NCT02908633. All patients enrolled in the pilot study and followed for 1 year were further observed for a prolonged time of 2 more years at 1-year intervals.

Assignment to one of three groups representing a type of C (ABeC, miniABeC, or ABiC) was generated randomly with a 1.0 allocation ratio on day 0. All patients received an ophthalmic examination at the preoperative stage (pre-washout). The presurgical period of withdrawal of antiglaucoma drugs took at least 30 days. Subjects were then controlled according to a scheme: day 0—1—7, month 1—3—6, and year 1—2—3. The exact details of data collection have been thoroughly described in the 1-year results paper [11].

Late complications that took place in the interim and at the 3-year follow-up were noted.

Two categories of surgical success, qualified and complete, were defined with IOP below 15 mmHg, medicated and unmedicated, respectively. Surgical failure was recognized when IOP rose over this level or a need for further antiglaucoma surgery emerged. Also, a criterion of a drop in IOP by 20% or more in relation to the post-washout period without medications was set [12].

The 24-2 tests were carried out with a Humphrey Field Analyzer (Carl Zeiss Meditec AG, Jena, Germany) to assess visual field (VF) defect severity at baseline, 6 months postoperatively, and after each year.

### 2.2. B Patients

Patient inclusion criteria involved cataract along with POAG at a mild or moderate stage [13] as well as ophthalmoscopic signs of glaucomatous optic neuropathy. Eligible patients were qualified for antiglaucoma surgery with concomitant cataract removal due to uncontrolled POAG.

The factors precluding patients from the study were typical for the C procedure: narrow or injured iridocorneal angle (ICA), previous ocular surgery or antiglaucoma laser treatment as well as secondary OAG. For the purpose of this study, any history of IOP above 30 mmHg was considered an excluding factor, since such IOP may close the collectors’ ostia [14]. Other possible causes of long-term VF loss were excluded, such as neurologic disorders, cranial trauma, intracranial neoplasms, other optic nerve neuropathies, or hereditary disorders.

**Figure 1:**
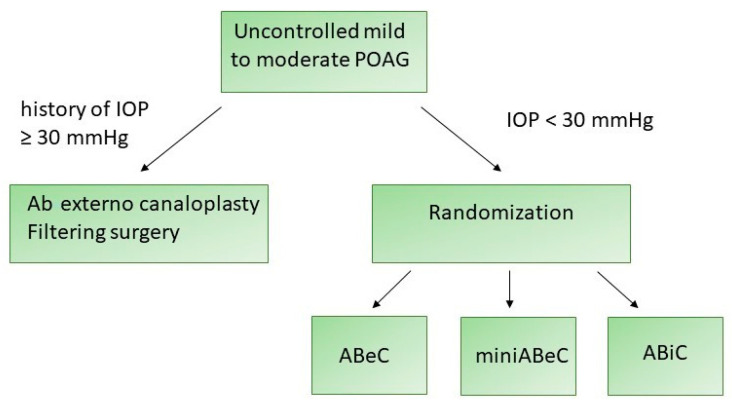
A flowchart presenting the management of the patients enrolled in this study.

The management of the patients is presented in the flowchart (see Figure 1).

### 2.3. Operative Technique

**Figure 2:**
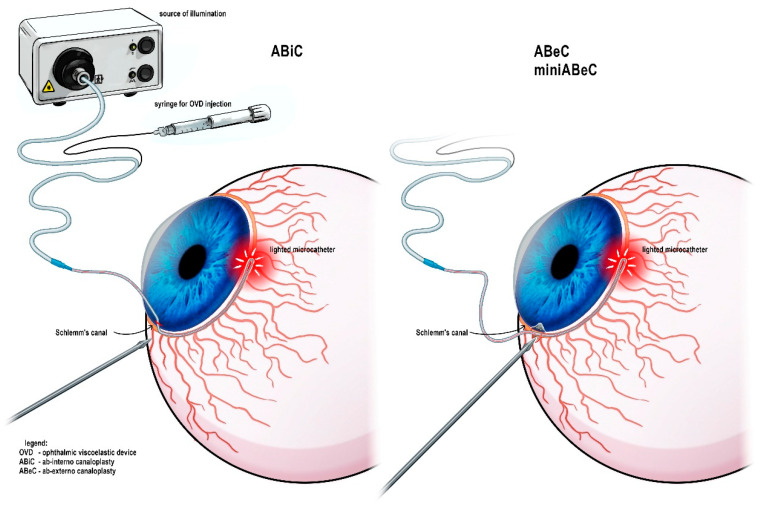
Instrumentation used during the canaloplasty procedure and variable possible access to the Schlemm’s canal.

The operative details have been presented in the earlier interim report [11]. One of the authors (M.R.) carried out all surgeries. In all cases, C was preceded by cataract phacoemulsification. For all surgeries, an iTrack (Nova Eye Inc., Fremont, CA, USA) with a diameter of 200 µm was used, and ophthalmic viscosurgical device (OVD) sodium hyaluronate (Healon GV, Johnson & Johnson Surgical Vision Inc., Santa Ana, CA, USA) was injected through its lumen into the SC every 2 hours. Both ABeC and miniABeC started with limbal peritomy in order to create a conjunctival flap with the base at fornix and scraping off of the vascular bed from the episclera. Afterwards, superficial and deep scleral flaps were created. In ABeC, the created flaps had the shape of a parabola, with dimensions of 5.0 × 5.0 mm for the superficial one and 4.5 × 4 mm for the deep one. Further dissection of the deep flap led to the exposure of the TDW and then excision. In miniABeC, the flaps were of different size; the upper one was thinner, rectangular, side wall-based, and 1 mm smaller in size, while the deeper one was only 1.0 × 1.0 mm in size and limbal-based. Afterwards, in both procedures, the SC was cannulated with iTrack and viscodilated, followed by a double 10.0 polypropylene suture placement. To seal the sclera, 10.0 Nylon was used in ABeC, while in miniABeC, none of the scleral flaps needed to be cut out; instead, they were just replaced and left unsutured after the catheterization of the SC was complete. At the conclusion of the surgeries, 8.0 absorbable sutures (ABeC) or cautery (miniABeC) were used for conjunctiva. ABiC, on the other hand, is a surgery classified as mini-invasive antiglaucoma surgery (MIGS), so it is tissue-sparing [15]. ABiC was performed with the use of a gonioscope. The intracameral placement of the iTrack was carried out via a clear corneal incision and advanced through the SC’s whole circumference. In ABiC, an OVD was injected into the SC similarly to ABeC, but without leaving a thread. The instrumentation and access to the SC is presented in Figure 2.

### 2.4. Statistics

To analyze the changes in IOP and CDVA in time for each group, Wilcoxon test was used (without Bonferroni correction [16]). The differences in levels of IOP and CDVA as well as in the number of antiglaucoma drops of particular types of surgery were analyzed pre-surgically and in various times throughout the follow-up period (Kruskal–Wallis). The survival function of both surgical successes was measured with the Kaplan–Meier estimator, while their comparison was performed with the log-rank test. The differences between the groups in terms of the number of patients meeting the additional criterion of 20% of IOP reduction was assessed with Fisher’s test. The α was established at 5%.

The R Statistical language (version 4.1.1; R Core Team, 2021) on Windows 10 ×64 (build 19044) was used as the analytics tool.

## 3. Results

All subjects were observed throughout the 2nd and 3nd year following the procedure, except for one—post ABeC—who died at 2 years. The patients’ demographic data were already published in the previous paper [11] and the VF severity is shown in Table 1. Table 2 shows the fluctuations of IOP and their reduction in relation to pre- and post-washout time (Wilcoxon).

### 3.1. IOP

**Figure 3:**
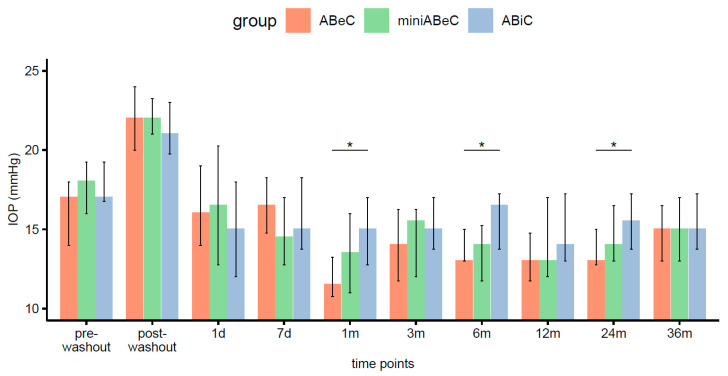
Distributions of intraocular pressure (medians and IQR, here and below) in all groups (ABeC, miniABeC, and ABiC) at each time point along with an estimate of the differences between the groups (notation of significance levels: *—*p* <0.050).

The withdrawal of topical medication caused an elevation of the median IOPs in each group (Wilcoxon). At two and three years postoperatively, all groups showed a lowering of pressure in a significant manner compared to the post-washout period (see Table 2 and Figure 3).

### 3.2. Comparison between Groups

We previously reported that the IOP’s medians only differed for ABeC and ABiC patients, at 1 (*p* = 0.022) and 6 months (*p* = 0.014) postoperatively [11]. Further observation revealed no statistically significant differences in the median intraocular pressure between various types of operations at 2 (*p* = 0.100) and 3 years (*p* = 0.810) after operation (Kruskal–Wallis, see Figure 3). The comparison of Kaplan–Meier plots showed the same probability of operational success for all surgeries in terms of both qualified and complete success at the 36-month follow-up (log-rank, *p* > 0.05). A decrease in IOP of 20% or more proved to be dependent on the type of group at the stage of 2 years postoperatively (Fisher’s test, *p* = 0.023). However, the ad hoc test with correction for multiple comparisons gave insignificant differences for the groups’ comparison. The percentage of unmedicated patients reaching a 20% drop in IOP after 2 years was higher in C compared to ABiC, but only at the level of a trend (*p* = 0.070). The application of Pearson’s chi-square test did not confirm this relation over three years (*p* = 0.519).

### 3.3. Number of Antiglaucoma Eye Drops

Table 3 illustrates the change in the number of antiglaucoma drops throughout the observation period. Two years postoperatively no eye drops were used in the ABeC group. Four post-ABiC patients required medications (range 1–3), meaning that 75% remained unmedicated. One patient after miniABeC required continuous intensification of treatment during the first year, ending with non-penetrating deep sclerectomy (NPDS) at 13 months. At 36 months postoperatively, post-ABiC patients stayed on the same number of medications, one post-ABeC patient and the reoperated post-NPDS patient required the introduction of one medication. This means that, at this stage, ~94% of patients after ABeC and miniABeC (each group), and 75% of patients after ABiC, remained unmedicated.

### 3.4. Surgical Success

**Figure 4:**
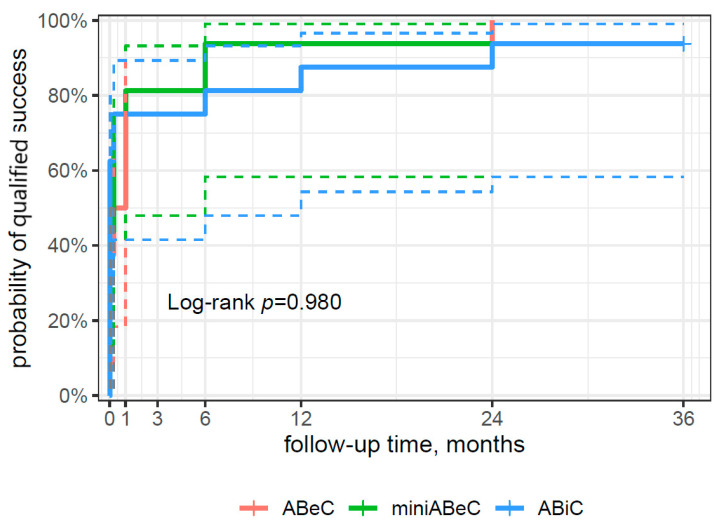
A plot of the Kaplan–Meier estimate of the survival function (risk type) of surgical success for patients undergoing ABeC, miniABeC, and ABiC with the log-rank test results (the solid lines of the appropriate color are the point value of 95% CI, and the dashed lines are the lower and upper limits of CI 95%).

**Figure 5:**
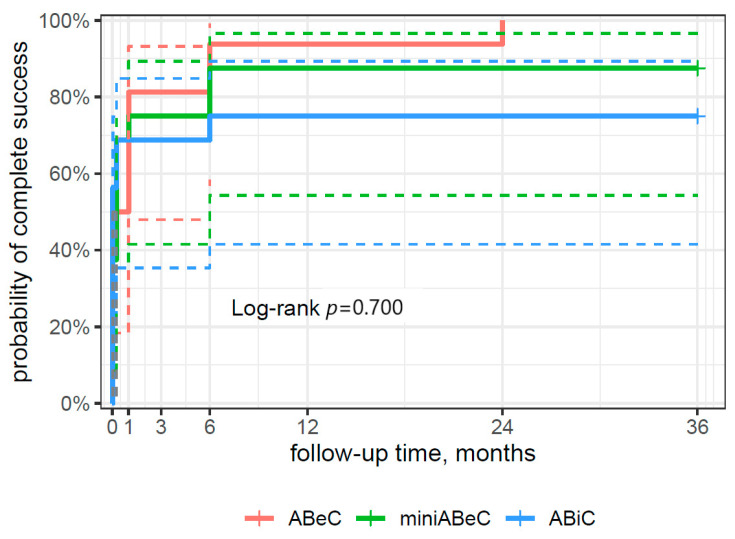
A plot of the Kaplan–Meier estimate of the survival function (risk type) of complete surgical success for the patients undergoing ABeC, miniABeC, and ABiC procedures with the log-rank test results (the solid lines of the appropriate color are the point value of 95% CI, and the dashed lines are the lower and upper limits of CI 95%).

From the survival curve statistics, the probability of qualified surgical success within three years after surgery was 94% for the ABiC and miniABeC groups, and 100% for the ABeC group (see Figure 4), and of complete surgical success, 75% and 100% for ABiC and miniABeC, respectively (see Figure 5). In patients not taking medication at 24 months postoperatively, a ≥20% reduction in IOP in relation to the post-washout stage was observed in *n* = 7 (43.8%) subjects post ABiC, *n* = 14 (87.5%) subjects post C, and *n* = 12 (75%) subjects post MC. Among patients not taking medication 36 months postoperatively, a 20% reduction in IOP was observed in *n* = 9 (56.2%) patients post ABiC, *n* = 12 (75.0%) patients post C, and *n* = 11 (68.8%) post MC.

### 3.5. Visual Acuity Results

**Figure 6:**
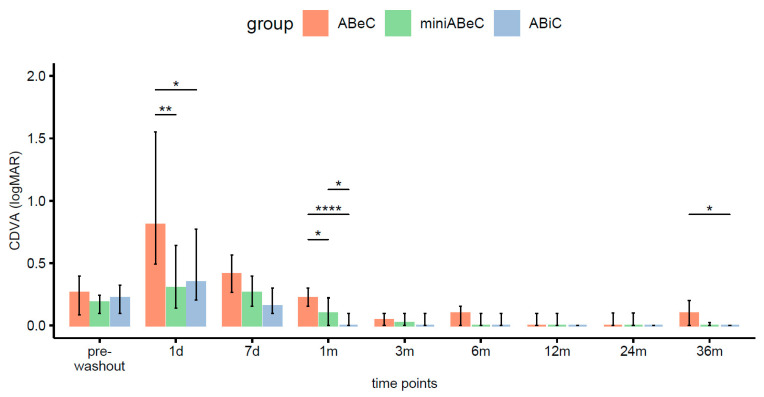
Distributions of CDVA (logMAR) in the ABeC, mini-ABeC, and ABiC groups at each time point along with an estimate of the differences between the groups (notation of the level of significant values: *—*p* < 0.050, **—*p* < 0.010, ****—*p* < 0.0001).

There were significant increases in CDVA (logMAR) compared to the pre-operative time point at 2 years, from 0.26 logMAR, 0.19 logMAR, and 0.22 logMAR in ABeC, miniABeC, and ABiC, respectively to 0.00 logMAR in all groups, and those results persisted until 36 months postoperatively. The CDVA showed no statistically significant differences between the three types of surgery at the 2-year follow-up stage. However, at 3 years postoperatively, this parameter differed significantly, when comparing the ABiC to the ABeC group in favor of the first one (Kruskal–Wallis test, see Figure 6).

### 3.6. Visual Fields

#### 3.6.1. MD

**Figure 7:**
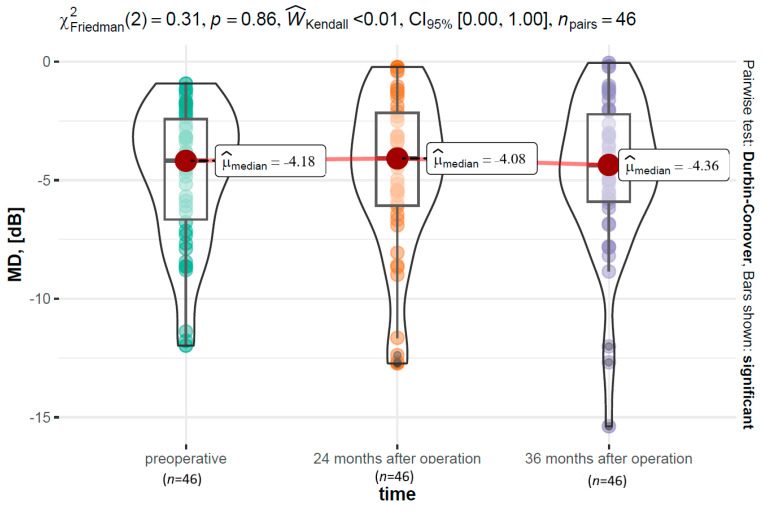
The MD parameter distribution over time, along with the results of a test examining within-group differences.

The analysis with the Kruskal–Wallis test showed that the MD parameter did not differ preoperatively between the ABiC (Mdn = −2.77 dB), C (Mdn = −4.68 dB), and MC groups (Mdn = −4.05 dB) (χ^2^ (2) = 3.24, *p* = 0.200, ϵ^2^ = 0.07). Two years postoperatively, the values of the MD parameter also did not differ between various surgeries (χ^2^ (2) = 5.64, *p* = 0.060, ϵ^2^ = 0.12). Similarly, no differences occurred 36 months postoperatively between the groups (χ^2^ (2) = 2.87, *p* = 0.240, ϵ^2^ = 0.24). Over time, namely, between the preoperative time point (Mdn = −4.18 dB), 24 months after operation (Mdn = −4.08 dB), and 36 months after operation (Mdn = −4.36 dB), there were no significant differences within the MD (Friedman ANOVA, χ^2^ (2) = 0.31, *p* = 0.860, ${\hat{W}}_{Kendall}$ < 0.01). A graphical visualization of the variable’s distribution along with the reporting of the results is presented in Figure 7.

#### 3.6.2. PSD

Similarly as for MD, the Kruskal–Wallis test revealed that the PSD values did not differ between the three types of C preoperatively (χ^2^ (2) = 3.00, *p* = 0.220, ϵ^2^ = 0.06) and at the stage of 2 years (χ^2^ (2) = 3.09, *p* = 0.210, ϵ^2^ = 0.07) or 3 years (χ^2^ (2) = 3.67, *p* = 0.160, ϵ^2^ = 0.08). PSD also remained stable throughout the observation period, and there were no significant differences within the PSD (Friedman ANOVA χ^2^ (2) = 1.1, *p* = 0.580, ${\hat{W}}_{\mathrm{Kendall}}$ = 0.01).

### 3.7. Incidence of Postsurgical Adverse Events

No intraoperative or early postoperative complications were reported. Typically for C, these would be a transient IOP increase or microhyphema/hyphema [11]. The only observed complication over a longer follow-up period was posterior capsule opacification (PCO) (see Table 4). At 2 years postoperatively, PCO occurred in three patients (one after ABiC and two after C), while at 36 months postoperatively, in only one patient—post ABiC. All patients were successfully treated by Nd:YAG laser capsulotomy.

## 4. Discussion

Despite being interesting, the results of this study should be treated as preliminary in their nature. The study has certain limitations, namely, a relatively small sample size and a short observation period. The scarce number of enrollees requires interpreting these results with great caution. In this trial, all modifications of C (ABeC, miniABeC, and ABiC) performed with phacoemulsification give a satisfactory lowering of pressure, a significant reduction in the number of medications, and an improvement of CDVA as well as VF stabilization without complications in Caucasians suffering from mild to moderate POAG. Two and three years postoperatively, no differences in median IOP reduction, achieved CDVA, or surgical success were noted. The only complication registered is PCO, which is rather a consequence of the phacoemulsification procedure and is easily removable. The content of this study is in line with previously published data on phacoC [17,18,19,20] and initial results of miniABeC [21]. This trial also adds to the state of the literature on ABiC. Three years after surgery, a few post-ABiC patients required reintroduction of topical treatment. Further research on a larger sample is needed to confirm this tendency. Perhaps ABiC would be more suitable for patients compliant to the usage of eye drops.

The promising outcomes of miniABeC also mean that it could serve as a less invasive alternative to the traditional procedure. In the authors’ opinion, its efficacy suggests that the scleral lake may be unnecessary, as the main IOP reduction results from the viscodilatation of the SC and the tensioning effect of the suture. The intrascleral space in NPDS becomes a reservoir of fluid between the filtration site and subconjunctival space, and provides more stable IOP reduction. Its role in C seems to be omittable, since it is a procedure focused on enhancing the distal outflow pathway.

Another interesting finding is the female gender predominance in all three groups. This is in general not in line with the results of meta-analyses [22]. A reason for that is worth considering. Some authors indicate the protective role of female hormones on the optic nerve and its reduced levels in the postmenopausal period [23].

### 4.1. Evolution of Non-Penetrating Glaucoma Surgery

Non-penetrating glaucoma surgery (NPGS) started in the 1950s and 1960s, when Epstein [24] and Krasnov [25] demonstrated their work. The first author noticed aqueous humor leakage when dissecting the pterygium from the corneal surface, which led him to perform a “deep sclerectomy” in glaucomatous patients. The second author invented the so-called “sinusotomy” procedure, in which the lumen of the SC was externalized via removal of a deep scleral block at one fourth of its circumference. Neither of these techniques won many followers because of the scarce availability of operating microscopes at the time. Meanwhile, classic TC arose and gained popularity because of its high efficacy and simplicity [26]. Interestingly, Cairns did not originally intend to perform scleral flap filtration surgery but only to remove part of the resistance located in the TM (which we now know would have not been successful when performed only on part of the ICA). However, he received a couple of blebless cases, presumably because of the removal of a part of the posterior TM together with the scleral spur, thus causing a cyclodialysis. It was only in the 1980s, when Zimmerman performed a surgery being a precursor to the NPDS, which he then named “nonpenetrating trabeculectomy”, with Fedorov [27] and Kozlov [28] modifying it further. The success of NPDS depends largely on the filtration of aqueous humor via the thin trabeculo-Descemet membrane (TDM), which allows its sufficient outflow at the site of maximum resistance [29]. This surgery requires some dexterity in order to perform a meticulous dissection in between Descemet’s membrane and the corneal stroma. A certain part of stromal tissue has to be removed in order to obtain proper filtration through the anterior part of the TM. The inner wall of the SC is pulled out, as well as the juxtacanalicular TM and SC endothelium. These maneuvers come from the so-called “ab externo trabeculectomy” [30,31], and the removed tissues were confirmed to be juxtacanalicular and partially corneoscleral TM in confocal microscopy.

### 4.2. Schlemm’s Canal Surgery

VC, proposed by Stegmann, emphasizes the role of an OVD (Healon GV) in supporting the SC’s ostial patency [1]. In this procedure, aqueous humor percolates through the TDM, but due to the tightly sutured superficial scleral flap, it can only enter the SC’s lumen and not the subconjunctival space. It can later flow circumferentially through the SC and via the collectors’ ostia into the aqueous veins, or alternatively, diffuse from the scleral space into the adjacent uveoscleral outflow track. In VC, the SC’s inner wall is not pulled off and the TM is not removed, but the viscoelastic may have a complex impact on these structures not only at the site of injection. In in vitro studies, viscoelastic was confirmed to disrupt the internal wall, endothelium, and internal structures between both walls of the SC, and thus bypass the main glaucomatous resistance, as well as to split the outer wall of the SC, and thus enhance uveoscleral outflow [32]. There is also the hypotheoretical role of viscoelastic as an antifibrotic and anti-inflammatory agent [33]. However, acute histological findings did not reveal a reflection in long-term outcomes of the procedure, which were disappointing [34,35,36].

C evolved from concepts elucidated in both viscocanalostomy and NPDS, with the advance of adding an angioplasty-inspired flexible microcatheter—iTrack. Throughout the years, its high safety and efficacy have drawn more attention, and SC surgery experienced a renaissance; however, its exact mechanism of action remains under debate. The normal outflow pathway is segmental because the circumferential flow within the SC is limited (probably due to either closed collector channels or adhesions between the SC’s walls).

Operating on the SC leaves its outer wall and structures distal to it intact, and thus distal resistance remains, which results in a higher IOP than would be the case if bypassing these structures directly into the subconjunctival space as in fistulizing procedures. This kind of resistance can even reach 40% of the total resistance, based on some research on enucleated healthy eyes [37]. Outflow via the SC is less important in NPDS, but crucial in VC and C.

C acts circumferentially and thus wins over 1 h trabeculotomy or YAG trabeculopuncture. What is more, except for viscodilatation, there is a permanently acting factor left in SC—the polypropylene suture. Manipulation of the SC in this procedure is hypothesized to cause breaks in its internal wall [32] or distend the TM in a so-called pilocarpine-like effect [38]. Catheterization and viscodilatation of the SC may also prevent or undo the collapse of its lumen. Even though there is no theoretical basis for proving that an expansion of the SC’s lumen will decrease IOP, some authors emphasize the role of the SC’s dimensions and morphology in outflow facility [39]. Nevertheless, viscoelastic is unlikely to stay in the SC for long enough to cause permanent changes. Also, it remains unknown whether the exact role of septae (which are disrupted during the procedure) is to cause or rather prevent the collapse of the SC’s lumen.

### 4.3. Transscleral Outflow in Canaloplasty and Mini-Canaloplasty

In NPDS, four ways of aqueous resorption after it passages the TDM have been hypothesized: subconjunctival bleb, intrascleral bleb, suprachoroidal outflow, and episcleral vein outflow via the SC [40].

In C, the watertight-closed superficial flap does not allow subconjunctival filtration. Suprachoroidal outflow in NPDS is not well documented but possible because of the ultra-thin residual sclera.

The exact importance of the intrascleral space remains unknown. This was the underlying concept behind miniABeC: to omit the time-consuming dissection of the TDM and remove small scleral blocks adjacent to the SC [21]. In NPDS, this structure makes for a reservoir of aqueous humor to prevent a large subconjunctival bleb. The outflow from the scleral lake is most probably thanks to the new drainage vessels [41]. Its volume, measured in ultrasound biomicroscopy (UBM), on the other hand, seemed not to matter in terms of IOP reduction after VC or NPDS [42,43]. The exact role of the intrascleral lake in C has not been extensively studied. Mastropasqua et al. emphasize the role of transscleral aqueous drainage, which they support by the increase of both the amount and area of epithelial microcysts in confocal microscopy of the superior bulbar conjunctiva in individuals after C with no bleb present [44]. According to the authors mentioned in the previous sentence, such cysts were similar to those described after successful TC [45,46,47]. Grieshaber et al. also support the importance of the intrascleral space in their study comparing two types of dissection in C, with the traditional scleral flap excision method resulting in lower IOPs [48]. Furthermore, the previously described IOP decrease post goniopuncture procedure without the presence of a filtering bleb [49] may provide further evidence in favor of transscleral filtration. The usual absence of a bleb after C and the watertight suturing of the superficial sclera should exclude the possibility of this drainage being a result of aqueous leakage between the scleral flaps further into the subconjunctival space. In our study, the IOP levels observed after miniABeC did not differ from those after ABeC. This may indicate the dominant role of the SC and TM distention and influence on the distal outflow pathway rather than intrascleral outflow. Our clinical results may suggest that miniABeC could serve as an alternative to the ABeC procedure.

Definitely, NPGS and SC surgery require more skill than TC, where only a block of corneoscleral tissue is removed. A meticulous layer-by-layer dissection of the aqueous outflow structures gives a thorough understanding of the outflow system’s dimensions and is critical for carrying out these procedures properly, and miniABeC is no exception. On the other hand, omitting the dissection of the TDM also eliminates serious complications such as its rupture and iris prolapse. The procedure should also be less time-consuming in experienced hands, since no deep flap excision or scleral or conjunctival sutures are required.

## 5. Conclusions

The purpose behind this investigation was to assess three modifications of C in terms of IOP-reducing potential and safety over a longer follow-up period. After 36 months of observation, all three variants were effective in decreasing IOP and medication numbers, with no late complications. The results revealed no significant differences while comparing the groups. Among patients after ABiC, a few needed medications; perhaps further observation on larger groups would enhance differences in medicated and unmedicated IOP reduction. In the authors’ opinion, all three modifications combined with phacoemulsification could be considered as adequate for individuals with POAG at a mild to moderate stage with vision-impairing cataract and no history of trauma to iridocorneal angle, and under the assumption of a patent outflow system. However, it needs to be stressed that this study is of a preliminary nature and has to be validated with more data from multicenter research.

In general, the efficacy of NPGS techniques in terms of IOP-reducing potential has been concluded to reach lower IOP levels than that of TC [50]. What is more, they are indicated rather as first-line treatment before chronic topical medications and IOP spikes distort the outflow pathway. For the aforementioned reasons, they will not replace the current gold standard, which is TC. However, traditional ABeC has a huge potential to develop in many ways. Its IOP-reducing potential may be enhanced by the use of antimetabolites [51] or deeper dissection to evoke suprachoroidal drainage [52]. Its microinvasive variant, ABiC, is an effective method with a clear corneal incision and total preservation of the conjunctiva. MiniABeC is a bridge procedure focusing on trabeculocanalicular outflow with a long-lasting suture effect, which spares time and tissues. The authors conclude that miniABeC can be an alternative to ABeC, which means that the dominant mechanism of action might be the restoration of the natural outflow pathway and pilocarpine-like effect rather than transscleral outflow or outflow via new intrascleral vessels.

## Figures and Tables

**Table 1:** Patients’ visual field severity. Ab externo canaloplasty (ABeC), mini-canaloplasty (miniABeC) and ab interno canaloplasty (ABiC).

Mean Deviation (MD) in dB	ABeC	MiniABeC	ABiC
Mild (MD < 6)			
*n* (%)	9 (56.3)	13 (81.3)	12 (75)
Moderate (MD 6–12) *n* (%)	7 (43.8)	3 (18.8)	4 (25)

**Table 2:** The values of IOP’s first quartiles (Q1), medians, third quartiles (Q3), and interquartile ranges (IQRs) during various follow-up times in particular groups (ab externo (ABeC), mini-canaloplasty (miniABeC), and ab interno canaloplasty (ABiC)), and the reduction in IOP at various periods of follow-up regarding the post-washout stage under *p* value * (Wilcoxon).

	ABeC		miniABeC		ABiC	
Time	IOP (mmHg) Q1, Median, Q3, IQR	*p* Value *	IOP (mmHg) Q1, Median, Q3, IQR	*p* Value *	IOP (mmHg) Q1, Median, Q3, IQR	*p* Value *
pre-washout	14.0, 17.0, 18.0, 4.0	<0.001	16.0, 18.0, 19.8, 3.8	0.001	16.3, 17.0, 19.8, 3.5	0.001
post-washout	20.0, 22.0, 24.0, 4.0	-	20.3, 22.0, 23.8, 3.5	-	19.3, 21.0, 23.0, 3.7	-
1 d	12.0, 16.0, 19.0, 7.0	0.008	12.3, 16.5, 20.8, 8.5	0.031	12.0, 15.0, 18.0, 6.0	0.003
7 d	14.3, 16.5, 18.8, 4.5	0.007	12.3, 14.5, 17.0, 4.7	<0.001	13.3, 15.0, 18.8, 5.5	<0.001
1 m	10.3, 11.5, 13.8, 3.5	<0.001	11.0, 13.5, 16.0, 5.0	<0.001	12.3, 15.0, 17.0, 4.7	<0.001
3 m	11.3, 14.0, 16.8, 5.5	<0.001	12.0, 15.5, 16.8, 4.8	0.001	13.3, 15.0, 17.0, 3.7	<0.001
6 m	13.0, 13.0, 15.0, 2.0	<0.001	11.3, 14.0, 15.8, 4.5	<0.001	13.3, 16.5, 17.8, 4.5	<0.001
12 m	11.3, 13.0, 16.3, 5.0	<0.001	12.0, 13.0, 17.0, 5.0	<0.001	13.0, 14.0, 17.8, 4.8	<0.001
24 m	12.8, 13.0, 15.0, 2.3	0.001	13.0, 14.0, 16.5, 3.5	0.010	13.8, 15.5, 17.3, 3.5	0.007
36 m	13.0, 15.0, 16.5, 3.5	0.001	13.0, 15.0, 17.0, 4.0	<0.001	13.8, 15.0, 17.3, 3.5	0.001

**Table 3:** Number of antiglaucoma eye drops (*n*) before the surgery and throughout the observation period in each group: ABeC, miniABeC, and ABiC.

Medications (*n*) Median (Range)			
Stage	ABeC	miniABeC	ABiC
pre-washout	2.0 (1 to 3)	2.0 (1 to 3)	2.0 (0 to 4)
post-washout	0	0	0
12 m	0	0 (0 to 4)	0 (0 to 3)
24 m	0	0	0 (0–3)
36 m	1 (0–1)	1 (0–1)	0 (0–3)

**Table 4:** Evaluation of postsurgical complications with the aid of the chi-square test.

Adverse Event	ABeC	MiniABeC	ABiC	*p* Value
PCO (posterior capsular opacification)	4/16	1/16	2/16	0.467

## Data Availability

The data presented in this study are available on request from the corresponding author. The data are not publicly available due to privacy restrictions of research participants.
